# Response surface optimization for recovery of polyphenols and carotenoids from leaves of *Centella asiatica* using an ethanol‐based solvent system

**DOI:** 10.1002/fsn3.832

**Published:** 2019-01-24

**Authors:** K. D. P. P. Gunathilake, K. K. D. S. Ranaweera, H. P. V. Rupasinghe

**Affiliations:** ^1^ Faculty of Livestock, Fisheries & Nutrition Department of Food Science & Technology Wayamba University of Sri Lanka, Makandura Gonawila Sri Lanka; ^2^ Faculty of Applied Sciences Department of Food Science and Technology University of Sri Jayewardenepura Gangodawila, Nugegoda Sri Lanka; ^3^ Faculty of Agriculture Department of Plant, Food, and Environmental Sciences Dalhousie University Truro Canada

**Keywords:** carotenoids, *Centella asiatica* leaves, phenolics, response surface methodology

## Abstract

Response surface methodology has been used to optimize the extraction conditions for total phenolics and carotenoids from leaves of *Centella asiatica*. Solvent concentration (30%–100%), extraction temperature (30–60°C), and extraction time (30–90 min) were used as the independent variables. A second‐order polynomial model produced a satisfactory fitting of the experimental data with regard to total phenolics (*R* ^2^ = 84.75%, *p* < 0.004) and carotenoid (*R* ^2^ = 78.74, *p* < 0.019) contents. The optimum extraction conditions of ethanol concentration, extraction temperature, and extraction time for phenolics were 6.1%, 70.2°C, and 110.5 min and for carotenoids, the optimum parameters were 100%, 70.2°C, and 110.5 min, respectively. The optimal predicted contents for total phenolics (9.03 mg Gallic Acid Equivalent (GAE)/g DW) and carotenoid (8.74 mg/g DW) values in the extracts were agreed with the experimental values obtained with optimum extraction conditions for each response, and also they possess significantly higher total antioxidant capacity.

## 1 INTRODUCTION

Recent studies indicate that phytochemicals such as polyphenols and carotenoids from numerous vegetables exert several health‐promoting functions, including reducing the risks of many chronic diseases such as certain types of cancer, cardiovascular, and neuro‐degenerative diseases (Vita, 2005). Most of these preventive effects of polyphenols and carotenoid compounds are associated with their antioxidant activity, protecting cells, and tissues from oxidative damage by various free radicals and reactive oxygen species (ROS) (Hayouni, Abedrabba, Bouix, & Hamdi, 2007; Sies & Stahl, 1995). Some of the recent trends in food and nutritional research are identifying antioxidant‐rich dietary sources, separation, and purification of bioactives from functional foods, and development of nutraceutical preparations.

*Centella asiatica* (Linn.) is a plant that belongs to the family Apiaceae, and the therapeutic use with its wide range of application has been documented. In many countries, leaves of *C*. *asiatica* are consumed as a leafy vegetable. Traditionally, *C. asiatica* has been valued for centuries in Ayurvedic medicine for the treatment of many illnesses such as asthma, bronchitis, elephantiasis, eczemas, anxiety, skin diseases, wound healing and for revitalizing the nerves, and brain cells, hence primarily known as a “Brain food” or “Memory enhancer” (Srivastava, Shukla, & Kumar, 1997). Photochemical analysis of *C. asiatica* plant extracts revealed the presence of various biochemical constituents such as polyphenols, carotenoids (Gunathilake & Ranaweera, 2016; Gunathilake, Ranaweera, & Rupasinghe, 2018), alkaloids, flavonoids, glycosides, triterpenoids, saponins, amino acids, inorganic acids, vitamins, sterols, and lipid compounds (Chippada & Vangalapati, 2011). Carotenoids are much effective antioxidant in scavenging singlet molecular oxygen and peroxyl radicals (Stahl & Sies, 2003), and Gunathilake and Ranaweera (2016) have reported that *C. asiatica* leaves possess antioxidant activities toward free radical scavenging, lipid peroxidation inhibition, and reducing potential. Previous studies have also shown that antioxidative activities of different parts of *C. asiatica* and the phenolic compounds have been suggested as the major contributors to the antioxidative and therapeutic activities (Zainol, Abd‐Hamid, Yusof, Muse, 2003). Further, it has been found that bioactive molecules present in *C. asiatica* can be used as active ingredients for the development of new drugs and natural health products (Pittella, Dutra, Junior, Lopes, Barbosa, 2009).

There is a current trend in investigating natural dietary sources of antioxidants such as green leafy vegetables for the formulation of value‐added functional food and nutraceutical ingredients. Extraction is the initial and most vital step in the recovery and purification of bioactive compounds from plant sources (Prasad et al., 2011). According to Gan and Latiff (2011), many factors such as solvent concentration, extraction temperature, solvent‐to‐solid ratio, and extraction duration may significantly influence the extraction efficiency and bioactive concentration (Gan & Latiff, 2011). Therefore, it is necessary to optimize the extraction conditions to obtain the highest bioactive recovery. There are investigations showing the antioxidant potential of *C. asiatica* but none of these explained the optimum extraction conditions for the extraction of bioactives for the use in nutraceutical or pharmaceutical applications. Response surface methodology (RSM) is a widely used tool to evaluate the effects of multiple factors and their interactions in one or more response variables. RSM, nowadays, is one of the most popular optimization techniques in the area of food science and technology and has been applied for the extraction of antioxidant bioactives from a number of dietary sources including *Zingiber officinale* (Gunathilake & Rupasinghe, 2014), olive leaves (Sahin & Samli, 2013), *Ipomoea batatas* leaves (Song, Li, Liu, & Zhang, 2011), *Brassica napus* (Wang & Liu, 2009), and *Inga edulis* leaves (Silva, Pompeu, Larondelle, & Rogez, 2007). There are no studies reported for the optimization of the extraction conditions for polyphenols and carotenoids from leaves of *C. asiatica*. Therefore, the objective of the present study was to investigate the optimum extraction conditions for *C. asiatica* leaves to obtain the highest polyphenols and carotenoid content. The findings would be much helpful for the functional foods and nutraceutical industries for the recovery of bioactives from this valuable herb.

## 2 MATERIAL AND METHODS

### 2.1 Plant materials

*Centella asiatica* leaves (type G_1_—”heen gotukola”) were collected from home gardens in Makandura area of Sri Lanka, and cleaned edible portions of this leaves were oven‐dried at 48°C for 48 hr, ground into powder using a blender, and were stored at −18°C until use. Voucher specimens of the samples have been deposited in the herbarium of the Department of Food Science and Technology of the Wayamba University of Sri Lanka.

### 2.2 Chemicals

Gallic acid and ethanol were purchased from Sigma‐Aldrich, St. Louis, MO, the USA, through Analytical Instrument Pvt Ltd, Colombo, Sri Lanka. All other chemicals used were of analytical grade.

### 2.3 Preparation of extracts

One gram of air‐dried and ground leaf sample was placed in a conical flask with 20 ml aqueous ethanol (1:20 solid/liquid ratio) at desired concentrations, and extraction was carried out for using a rotary shaker (Unimax 1010; Heidolph, Kelheim, Germany) at 400 rpm, at specified temperature as dictated by the experimental design. The response surface optimization procedure was designed based on a three‐factor inscribed central composite design (CCD) consisting of aqueous ethanol (30%–100%), extraction temperature (30–60°C), and extraction time (30–90 min) as shown in Table 1. The extracts were then filtered through a filter paper (Whatman No. 42; Whatman Paper Ltd, Maidstone, UK), and the filtrates were stored at −18°C until used for the determination of total polyphenols and carotenoid contents.

**Table 1:** Levels of extraction variables for experimental designs

Independent variables	Level total polyphenol and carotenoid contents				
	+1	0	−1	+1.682	−1.682
X1: Ethanol (%)	100	65	30	123.86	6.137
X2: Temperature (°C)	60	45	30	70.23	19.773
X3: Time (min)	90	60	30	110.45	9.546

### 2.4 Determination of total polyphenol content

The total polyphenol content was determined using Folin–Ciocalteu assay (Singleton, Orthofer, & Lamuela‐Raventos, 1999) with some modification, as described by Gunathilake, Yu, and Rupasinghe (2014) and Gunathilake (2012). About 0.5 ml of leaf extract and 0.1 ml of Folin–Ciocalteu reagent (0.5N) were mixed and incubated at room temperature (30°C) for 15 min at dark. Sodium carbonate (7.5%, 25 ml) was added and incubated for further 2 hr at dark. Absorbance was measured at 760 nm using UV/VIS spectrometer (Optima, SP‐3000, and Tokyo, Japan). Gallic acid was used to prepare standard curve, and the concentration of total polyphenols was expressed as mg of gallic acid equivalents (GAE) per gram dry weight.

### 2.5 Total carotenoid content

The carotenoid content was analyzed according to the method described by Şükran, Gunes, and Sivaci (1998) with slight modifications, and carotenoid contents were reported as mg/g DW. According to this method, total carotenoids were determined after having subtracted the concentration of chlorophyll A and B, using wavelengths 662 and 645 nm, respectively, and corresponding absorption coefficients at which carotenoids do not absorb.

### 2.6 Determination of total antioxidant capacity

The total antioxidant capacity of leaf extracts was analyzed according to the method described by Prieto, Pineda, and Aguilar (1999) with some modifications of Gunathilake and Ranaweera (2016). Briefly, 0.3 ml leaf extract and 3 ml reagent solution (0.6 M sulphuric acid, 28 mM sodium phosphate, and 4 mM ammonium molybdate) were incubated at 95°C for 90 min, and then, the solution was cooled to room temperature (30 ± 2°C), and the absorbance of each solution was measured at 695 nm spectrophotometrically against a blank. The antioxidant capacity was expressed as ascorbic acid equivalents (AAE).

### 2.7 Experimental design

Optimization of extraction parameters of phenolics from *C. asiatica* leaves was done using RSM.

Influence of three independent variables, ethanol concentration, extraction temperature, and extraction time and the response variables were total phenolic, and total carotenoid contents were studied. A three‐factor inscribed CCD was used to identify the relationship existing between the response functions and the process variables, as well as to determine those conditions that optimized the extraction process of total phenolics and carotenoid contents of the extracts. The independent variables and the range studied were ethanol concentration (30–100%), temperature (30–60°C), and extraction time (30–90 min), and solid‐to‐liquid ratio was maintained at 1:20. The selection and range of these three factors were based on previous studies. Each variable to be optimized was coded at three levels 1, 0, +1 (Table 1). According to the design used, twenty randomized experiments including six replicates as the center points were assigned based on CCD and the values of independent process variables considered, as well as measured total phenolic content and carotenoid content, are given in Table 2.

**Table 2:** Central composite design arrangement for extraction of polyphenols and carotenoids from *Centella asiatica*

Run order	Ethanol%	Temperature (°C)	Time (min)	Polyphenols mg/g DW	Carotenoids mg/g DW
1	30	60	90	4.13	0.71
2	123.9	45	60	1.43	2.44
3	65	45	9.5	3.06	1.93
4	30	30	90	3.64	0.73
5	65	45	60	3.43	2.17
6	65	45	60	3.99	2.37
7	65	45	60	2.27	3.64
8	65	19.8	60	4.31	2.47
9	100	30	30	1.54	2.45
10	65	45	110.5	3.72	2.75
11	65	45	60	3.24	2.76
12	30	60	30	3.57	1.11
13	30	30	30	3.31	0.98
14	100	30	90	1.61	2.57
15	65	45	60	3.38	2.5
16	65	70.2	60	4.09	2.71
17	6.1	45	60	2.98	0.89
18	100	60	90	2.24	3.47
19	100	60	30	1.65	2.61
20	65	45	60	3.42	2.23

### 2.8 Statistical design

Acquired data were handled to calculate statistical values such as mean and standard deviation (*SD*) using Microsoft Excel (Microsoft Inc., Redmond, WA, USA). For data analysis, Minitab 15 software was used. The assumptions of normality and constant variance were checked and confirmed. A response surface analysis and analysis of variance (ANOVA) were employed to determine the regression coefficients, the statistical significance of the model terms, and to fit the mathematical models of the experimental data that aimed to optimize the overall region for both response variables. A second‐order polynomial model was used to predict the response variables as appeared below:

$$\begin{array}{cc} Y=\;\; & \beta_{0}+\beta_{1}X_{1}+\beta_{2}X_{2}+\beta_{3}X_{3}+\beta_{1}^{2}X_{1}^{2}+\beta_{2}^{2}X_{2}^{2}+\beta_{3}^{2}X_{3}^{2}+\beta_{1}\beta_{2}X_{1}X_{2}+\\ & \beta_{1}\beta_{3}X_{1}X_{3}+\beta_{2}\beta_{3}X_{2}X_{3} \end{array}$$

where Y is the predicted dependent variable; β_0_ is a constant that fixes the response at the central point of the experiment; β_1_, β_2_, and β_3_ are the regression coefficients for the linear effect terms; $\beta_{1}^{2}$, $\beta_{2}^{2}$, and $\beta_{3}^{2}$ are the quadratic effect terms; and β_1_β_2_, β_1_β_3_, and β_2_β_3_ are the interaction effect terms, respectively. X1, X2, and X3 are the independent variables (Table 1). The adequacy of the model was predicted through the regression analysis (*R* ^*2*^) and the ANOVA analysis. The relationship between the independent variables and the response variables (polyhenols and carotenoids) was demonstrated by the response surface plots. Multiple graphical and numerical optimizations of the experimental data were done to identify the optimum extraction conditions to achieve the maximum recovery of polyphenols and carotenoids. Verification of predicted extraction conditions that would give higher levels of polyphenols and carotenoids was determined based on the best extractions conditions obtained with RSM.

## 3 RESULTS AND DISCUSSION

Twelve morphotypes of *C. asiatica* have been recorded according to their morphological and morphometric characters in Sri Lanka and among the types available, types G1 and G2 known as “heen gotukola” which is having smaller leaves and are more popular among the local community as highly nutritious types. Types G8 and G12 known as “giant gotukola” have very large leaves, and they contained higher amount of β‐carotene and lutein (Chandrika, Salim, Wijepala, Perera, & Goonetilleke, 2011). The presence of various phenolic and carotenoid bioactives such as triterpene saponins, asiaticoside, numerous caffeic acid derivatives, and flavonoids in *C. asiatica* is believed to be responsible for health benefits associated with this leafy vegetable (Chippada & Vangalapati, 2011).

An optimization of extraction conditions for the recovery of total polyphenols and carotenoids from *C. asiatica* was conducted using RSM. The extraction efficiency of these bioactive constituents was influenced by extraction solvent properties, extraction time, and extraction temperature (Alothman, Bhat, & Karim, 2009). RSM is considered as a powerful tool in optimizing experimental conditions to maximize various responses (Hajj, Louka, Nguyen, & Maroun, 2012). Leaves of *C. asiatica* contain polyphenols, carotenoids and possess antioxidant activity (Gunathilake & Ranaweera, 2016; Rahman et al., 2013). For the studies on optimization of extraction of bioactive molecules, extraction is one of the most vital steps in the recovery and purification of bioactives from potential dietary sources. The efficiency and effectiveness of the polyphenol and carotenoid extraction process are generally influenced by multiple variables, including solid‐to‐solvent ratio, extraction time, temperature, and solvent composition (Alothman et al., 2009). The uncoded coefficient values for the experimental designs for total polyphenols and carotenoids of *C. asiatica* leaves are given in Table 2. The obtained data were used for the prediction of an optimum set of extraction parameters from the leaf extract with high polyphenols and carotenoids. The concentration of polyphenols and carotenoids in the extracts was employed in a multiple regression analysis, performed using RSM to fit the second‐order polynomial equations is given in Table 3 for polyphenols and carotenoids, respectively. The “fitness” of the model was studied through the lack‐of‐fit test (*p* ≤ 0.05), which indicated the adequacy of models to accurately predict the variation (Kong, Ismail, Tan, Prasad, & Ismail, 2010). The quality of fit to the second‐order polynomial models for leaf extracts of *C. asiatica* was established based on the coefficients of determination (70%> *R* ^2^), regression *p*‐value (*p* ≤ 0.1), and lack of fit (*p* ≤ 0.05) indicating that the models could be used to predict the responses. The software generated the estimated regressions coefficients for quadratic equations as appeared in Table 3.

**Table 3:** Estimated regression coefficients of the second‐order polynomial equations for RSM analysis of total polyphenols and carotenoid extraction (uncoded) from *Centella asiatica*

Polyphenols terms	Regression coefficients	Probability	Regression *p*‐value	*R* ^2^	Lack of fit
Constant	4.31512	0.000	0.020	78.35%	0.402
Ethanol %	0.0333913	0.001			
Temperature (°C)	−0.0891847	0.623			
Time (min)	0.00964536	0.256			
Ethanol %*Ethanol %	−4.07814E‐04	0.010			
Temperature *Temperature (°C)	040.000914520	0.220			
Time (min)*Time (min)	−8.95681E‐05‐	0.620			
Ethanol %*Temperature (°C)	2.38095E‐06‐	0.995			
Ethanol %*Time (min)	2.73810E‐	0.864			
Temperature (°C)*Time (min)	050.000208333	0.667			
Carotenoids					
Constant	−0.299526	0.000	0.017	79.28%	0.523
Ethanol %	0.0427033	0.000			
Temperature (°C)	0.0152902	0.448			
Time (min)	0.00668319	0.411			
Ethanol %*Ethanol %	−3.32569E‐04	0.017			
Temperature*Temperature (°C)	−3.57157E‐04	0.584			
Time (min)*Time (min)	−1.87499E‐04	0.262			
Ethanol %*Temperature (°C)	0.000226190	0.547			
Ethanol %*Time (min)	0.000194048	0.310			
Temperature (°C)*Time (min)	0.000163889	0.707			

### 3.1 Model fitting of parameters based on total phenolic and carotenoid content

For RSM, the levels of independent variables for the extraction of total polyphenols and carotenoids were selected based on the literature. The experimental design and corresponding response data are presented in Table 2. Total polyphenol content of leaf extracts varied from 1.43 to 4.31 mg GAE/g dry sample. Total carotenoid contents varied from 0.71 to 3.64 mg/g DW. The ANOVA of the second‐order polynomial models for the polyphenol extractions from *C. asiatica* leaves shows that the models were significant (*p* ≤ 0.05) with *R* ^2^ and *p*‐values of 0.78 and 0.02, respectively (Table 3). There was no significance in the lack of fit (*p* = 0.40) in the model indicating that the model could be used to predict the responses. The quadratic regression models for carotenoid extraction showed that the models were significant (*p* < 0.05) with *R* ^2^ and *p*‐values of 0.79 and 0.017, respectively (Table 3). The lack of fit (*p* = 0.52) in the model was not significance (*p* < 0.05), and this indicated that the model could be used to predict responses. To visualize the relationship between the response and experimental levels of the independent variables for the total phenolics and carotenoid extraction, three‐dimensional (3D) surface plots were constructed according to the quadratic polynomial model equations of Table 3.

### 3.2 Effect of extraction parameters on total phenolic content

**Figure 1:**
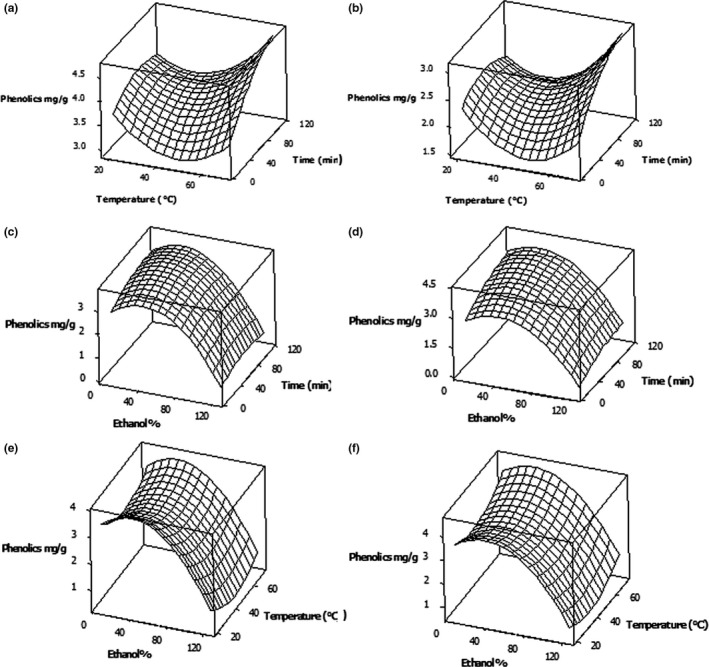
Pair wise response surface plots of polyphenol (mg GAE/g DW) extraction from *Centella asiatica* leaves as a function of ethanol %, extraction temperature, and time: Ethanol % was kept constant at 30% (a) and 100% (b); temperature of extraction was kept constant at 30°C (c) and 60°C (d); and the time of extraction was kept constant at 30 min (e) and 90 min (f)

**Figure 2:**
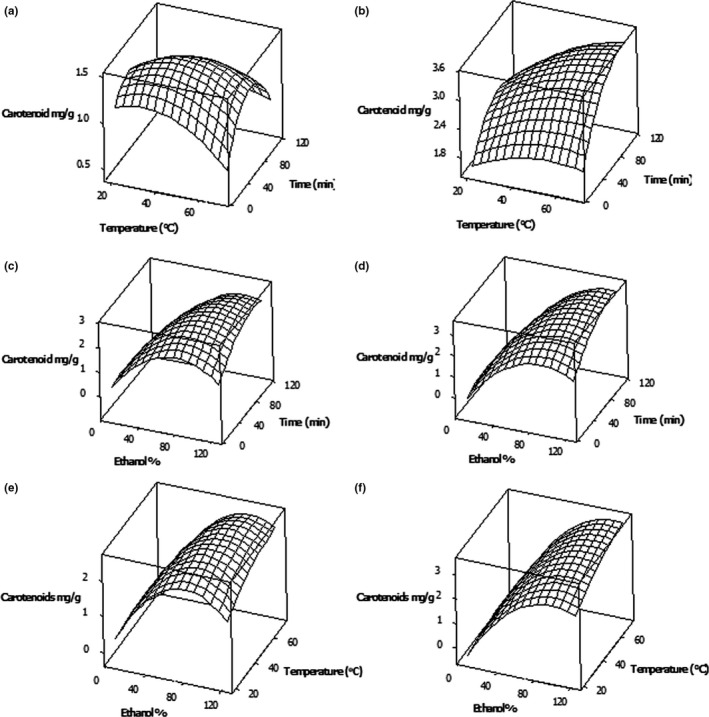
Pair wise response surface plots of the carotenoid (mg/g DW) extraction from *Centella asiatica* leaves as a function of ethanol %, extraction temperature, and time: Ethanol % was kept constant at 30% (a) and 100% (b); temperature of extraction was kept constant at 30°C (c) and 60°C (d); and the time of extraction was kept constant at 30 min (e) and 90 min (f)

Response surfaces were used to illustrate the effects of solvent concentration, extraction time, and the temperature on the responses (Figures 1 and 2). The responses demonstrated that the ethanol concentration, extraction temperature, and the duration of the extraction greatly affect the recovery of polyphenols from *C. asiatica* leaves (Figure 1). The type of solvent plays an important role in the extraction of polyphenols and other antioxidant compounds from complex biological materials such as leafy vegetables. Further, the use of ethanol is relatively cheap, reusable, and nontoxic organic solvent and could lend an environmentally friendly aspect to the low‐cost preparation of potentially bioactive extracts for food and nutraceutical uses. Therefore, many researchers have used aqueous ethanol for the extraction of various bioactive antioxidants from plants sources when used for food uses (Hayouni et al., 2007; Tabaraki & Nateghi, 2011).

Based on the results, ethanol concentration had a curved relationship with polyphenol extraction.

While lower ethanol % as extraction solvent were distinguished by the highest level of extract yield of polyphenols, pure ethanol (100% ethanol) showed the poorest level of all the other solvent systems used for *C. asiatica* (Figure 1). As the extraction and isolation of polyphenols depend greatly on the polarity of the extraction solvent, the use of a pure solvent may not be effective for the separation of polyphenols from plant materials as described in Prasad et al. (2011). Further, according to Lang and Wai (2006), water is acting as the plant swelling agent, while ethanol may disrupt the bonding between the solutes and plant matrices. This is indicating that the mixture of water and ethanol or “aqueous ethanol” as solvent agent exhibited the best performance to extract polyphenols from plant sources. Therefore, a combination of alcohol with water seems more effective in extracting polyphenols. This is consistent with several earlier findings which convey that polyphenols are more extractable in polar solvents as compared to non‐polar ones (Hayouni et al., 2007; Prasad et al., 2011).

Regarding extraction temperature on total polyphenols, the recovery of phenolics was increased considerably when the extraction temperature was increased to 60°C, while the % ethanol maintained at a low level (Figure 1c and d). Results showed that at lower solvent concentration (30%), the use of higher extraction temperature (60°C) and extraction time (90 min) increased the extractable phenolics from 3.31 to 4.13 mg GAE/g DW, compared with the use of lower extraction temperature (30°C) and extraction time (30 min). This could be due to the increase in the solubility of polyphenols, diffusion rate, mass transfer rate, extraction rate, and reduced solvent viscosity and surface tension at higher temperatures and solvent polarities which could improve the polyphenol extractability (Richter et al., 1996). The extraction time was another important parameter in the extraction procedure for bioactives in many previous studies (Gunathilake & Rupasinghe, 2014; Sahin & Samli, 2013). However, the results showed that extraction time did not have a significant effect on the polyphenol extraction from *C. asiatica* leaves at *p* ≤ 0.05 level.

### 3.3 Effect of extraction parameters on carotenoid content

Various solvent systems have been used for carotenoid extraction, and ethanol is also a good solvent that can be used for carotenoid extraction (Ofori‐Boateng & Lee, 2013) and the extraction is highly influenced by extractions variables including solvent concentration, extraction temperature, and time (Wang & Liu, 2009). Many researchers have used anon‐polar solvent for carotenoid extraction, petroleum ether/acetone (1/1) for rapeseed (Wang & Liu, 2009), and hexane/acetone/alcohol (2/1/1) for lycopene (Kaur, Wani, Oberoi, & Sogi, 2008). Influence of three extraction conditions toward total carotenoid extraction was reported with the coefficients of the second‐order polynomial regression equation in Table 4. Results showed that the extraction of carotenoids had a greater influence of ethanol concentration and was significant (*p* ≤ 0.05). The extraction and separation of carotenoids depend largely on the nature of the polarity of the solvents (Wang & Liu, 2009). For *C. asiatica*, higher carotenoid extractions were observed when 100% ethanol was used (Figure 2) indicating it prefers toward non‐polar conditions. When ethanol concentration increased from 30% to 100% while keeping extraction temperature and time at 30°C and 30 min, respectively, increase in the carotenoid content from 0.98 to 2.45 mg/g DW was observed (Table 2). This may be due to the presence of more non‐polar carotenoids in *C. asiatica*. Rahman et al. (2013) also reported similar pattern where the extractable carotenoid content of *C. asiatica* leaves increases when ethanol concentration increased and they have reported higher total carotenoid content (1.1 mg/g) in *C. asiatica* leaves when 100% ethanol was used in comparison with 50% ethanol (0.70 mg/g).

**Table 4:** Predicted values and experimental values of total polyphenols and carotenoids at the optimum extraction conditions

Optimum extraction conditions		Predicted values (mg/g)		Experimental values (mg/g)	
Polyhenols	Carotenoids	Polyphenols	Carotenoids	Polyphenols	Carotenoids
ETOH: 37%	ETOH: 100%	4.71	3.55	4.58 ± 0.44	3.28 ± 0.39
Temp: 70.2 °C	Temp: 70.2°C				
Time: 110.5 min	Time: 110.5 min				

Extraction temperature and extraction duration showed the impact on carotenoids in many research (Mele′ndez‐Martı′nez, George, & Isabel, 2007); Ofori‐Boateng & Lee, 2013). In our study, when the extraction temperature increased from 30 to 60°C, while keeping the solvent concentration and extraction time at 100% and 30 min, respectively, a slight increase in the carotenoid content from 2.45 to 2.61 mg/g DW was observed (Figure 2c and 2d). Though Mele′ndez‐Martı′nez et al. (2007) have reported that carotenoids are degraded at elevated temperatures, conventional maceration, and Soxhlet extraction require high temperatures (over 70°C) for optimal carotenoid yields unless used ultrasonic like advanced technology (Guo, Zou, & Sun, 2010). This could be due to the releasing of more carotenoids from plant tissues because of greater disruption to the cell walls at a higher temperature. However, higher carotenoid content (3.64 mg/g DW) was observed in the extract which has extracted with 65% ethanolic concentration at 45°C temperature for 60‐min duration. Extraction time showed a significant effect on the carotenoid extraction from *C. asiatica* leaves at *p* ≤ 0.05 level. An increase in extractable carotenoid content from 2.61 to 3.47 mg/g DW was observed when the extraction time increased from 30 to 90 min while keeping solvent concentration and extraction temperature at 100% and 60°C, respectively.

### 3.4 Optimization of polyphenols and carotenoids and verification of the model

Optimum process parameters achieved by maximizing total phenolics and carotenoid contents. During the optimization stage, the desirability function of the MINITAB statistical software is used to obtain the best compromise of the two responses with the weights of all 1.0. As shown in Table 4, the predicted optimal ethanol concentration, extraction temperature and extraction time were developed for maximizing the both responses, and they were 37%, 70.20°C, and 110.5 min for phenolics and 100%, 70.20°C, and 110.5 min for carotenoids, respectively. For these optimum extraction conditions, the corresponding predicted response values for phenolics and carotenoids were 4.71 mg GAE/g DW and 3.55 mg/g DW, respectively. An experiment was run by the recommended optimum conditions for two responses, phenolics, and carotenoids. More interestingly, in this study, the values obtained experimentally for both response variables are near to the predicted values, indicating a satisfactory model. The experimental values for total phenolics were 4.58 ± 0.44 mg GAE g extract and 3.28 ± 0.39 mg/g DW carotenoids, and no significant difference (*p* < 0.05) was found between the experimental and predicted values of the extractable phenolics and carotenoids from leaves of *C. asiatica* extract. Further, the extracts prepared with the optimum extraction conditions showed significantly higher (*p* < 0.05) total antioxidant capacity compared with the extract prepared with 100% ethanol, 30°C, and 30 min extraction conditions. Therefore, the data confirm the validity of the optimized model.

## 4 CONCLUSIONS

An ethanol‐based extraction technique was applied for the extraction of polyphenols and carotenoid compounds from *C. asiatica* leaves and optimized by response surface methodology. The results showed that the extraction conditions including solvent concentration, extraction temperature, and extraction time markedly influenced the yields of total phenolics and total carotenoids of the *C. asiatica* extracts. Overall, extraction of polyphenols prefers low ethanol concentration, higher temperature, and longer extraction time, whereas higher carotenoid recovery was observed at higher ethanol concentrations and low temperatures. The optimum extraction conditions for polyphenols were as follows: ethanol concentration 37%, extraction temperature 70.20°C, and extraction time 110.5 min, which resulted in 4.71 mg/g DW total polyphenols. For carotenoids, optimum extraction conditions were ethanol concentration 100%, extraction temperature 70.20°C, and extraction time 110.5 min, which yield 3.55 mg/g DW carotenoids. It was confirmed that the predicted total phenolics and carotenoid content not significantly different with those of experimented values.

## CONFLICT OF INTEREST

The authors declare that they do not have any conflict of interest.

## ETHICAL REVIEW

This study does not involve any human or animal testing.

## References

[fsn3832-bib-0001] Alothman, M. , Bhat, R. , & Karim, A. A. (2009). Antioxidant capacity and phenolic content of selected tropical fruits from Malaysia, extracted with different solvents. Food Chemistry, 115, 785–788. 10.1016/j.foodchem.2008.12.005

[fsn3832-bib-0002] Chandrika, U. G. , Salim, N. , Wijepala, G. D. D. J. , Perera, K. S. U. , & Goonetilleke, A. K. E. (2011). Carotenoid and mineral content of different morphotypes of *Centella asiatica* L. (Gotukola). International Journal of Food Sciences and Nutrition, 62(5), 552–557. 10.3109/09637486.2011.552485 21391792

[fsn3832-bib-0003] Chippada, S. C. , & Vangalapati, M. (2011). Antioxidant, an anti‐inflammatory and anti‐arthritic activity of *Centella asiatica* extracts. Journal of Chemical, Biological and Physical Sciences, 1(2), 260.

[fsn3832-bib-0004] Gan, C. , & Latiff, A. A. (2011). Optimisation of the solvent extraction of bioactive compounds from *Parkia species* pod using response surface methodology. Food Chemistry, 124, 1277–1283. 10.1016/j.foodchem.2010.07.074

[fsn3832-bib-0005] Gunathilake, K. D. P. P. (2012). A fruit‐based functional beverage designed to reduce the risk of cardiovascular disease. MSc thesis. Dalhousie University; Halifax, NS, Canada.

[fsn3832-bib-0006] Gunathilake, K. D. P. P. , & Ranaweera, K. K. D. S. (2016). Antioxidative properties of 34 green leafy vegetables. Journal of Functional Foods, 26, 176–186. 10.1016/j.jff.2016.07.015

[fsn3832-bib-0007] Gunathilake, K. D. P. P. , Ranaweera, K. K. D. S. , & Rupasinghe, H. P. V. (2018). Change of phenolics, carotenoids, and antioxidant capacity following simulated gastrointestinal digestion and dialysis of selected edible green leaves. Food Chemistry, 245(15), 371–379. 10.1016/j.foodchem.2017.10.096 29287383

[fsn3832-bib-0008] Gunathilake, K. D. P. P. , & Rupasinghe, H. P. V. (2014). Optimization of water based‐extraction methods for the preparation of bioactive‐rich ginger extract using response surface methodology. European Journal of Medicinal Plants, 4(8), 893 10.9734/EJMP

[fsn3832-bib-0009] Gunathilake, K. D. P. P. , Yu, L. J. , & Rupasinghe, H. P. V. (2014). Reverse osmosis as a potential technique to improve antioxidant properties of fruit juices used for functional beverages. Food Chemistry, 148, 335–341. 10.1016/j.foodchem.2013.10.061 24262566

[fsn3832-bib-0010] Guo, X. , Zou, X. , & Sun, M. (2010). Optimization of extraction process by response surface methodology and preliminary characterization of polysaccharides from *Phellinus igniarius* . Carbohydrate Polymers, 80, 344–349. 10.1016/j.carbpol.2009.11.028

[fsn3832-bib-0011] Hajj, Y. E. , Louka, N. , Nguyen, C. , & Maroun, R. G. (2012). Low‐cost process for phenolic compounds extraction from cabernet sauvignon grapes (*Vitisvinifera* L. cv. *cabernet sauvignon*). Optimization by response surface methodology. Food and Nutrition Sciences, 3, 89–103. 10.4236/fns.2012.31014

[fsn3832-bib-0012] Hayouni, E. A. , Abedrabba, M. , Bouix, M. , & Hamdi, M. (2007). The effects of solvents and extraction method on the phenolic contents and biological activities in vitro of Tunisian *Quercuscoccifera* L and *Juniperusphoenicea* L. fruit extracts. Food Chemistry, 105, 1126–1134. 10.1016/j.foodchem.2007.02.010

[fsn3832-bib-0013] Kaur, D. , Wani, A. A. , Oberoi, D. P. S. , & Sogi, D. S. (2008). Effect of extraction conditions on lycopene extractions from tomato processing waste skin using response surface methodology. Food Chemistry, 108(2), 711–718. 10.1016/j.foodchem.2007.11.002 26059152

[fsn3832-bib-0014] Kong, K. W. , Ismail, A. R. , Tan, S. T. , Prasad, K. M. N. , & Ismail, A. (2010). Response surface optimisation for the extraction of phenolics and flavonoids from a pink guava puree industrial by‐product. International Journal of Food Science & Technology, 45, 1739–1745. 10.1111/j.1365-2621.2010.02335.x

[fsn3832-bib-0015] Lang, Q. , & Wai, C. M. (2006). Recent advances in extraction of nutraceuticals from plants. Trends in Food Science and Technology, 17, 300–312.

[fsn3832-bib-0016] Mele′ndez‐Martı′nez, J. A. , George, B. , & Isabel, M. V. (2007). Relationship between the colour and the chemical structure of carotenoid pigments. Food Chemistry, 101, 1145–1150. 10.1016/j.foodchem.2006.03.015

[fsn3832-bib-0017] Ofori‐Boateng, C. , & Lee, K. T. (2013). Response surface optimization of ultrasonic‐assisted extraction of carotenoids from oil palm (*Elaeis guineensis* Jacq.) fronds. Food Sciences and Nutrition, 1(3), 209–221. 10.1002/fsn3.22 PMC5779322 29387349

[fsn3832-bib-0140] Pittella, F. , Dutra, R. C. , Junior, D. D. , Lopes, M. T. , & Barbosa, N. R. (2009). Antioxidant and cytotoxic activities of *Centella asiatica* (L) Urb. International Journal of Molecular Sciences, 10, 3713–3721. 19865514 10.3390/ijms10093713 PMC2769141

[fsn3832-bib-0019] Prasad, K. N. , Hassan, F. A. , Yang, B. , Kong, K. W. , Ramanan, R. N. , Azlan, A. , & Ismail, A. (2011). Response surface optimisation for the extraction of phenolic compounds and antioxidant capacities of underutilized *Mangifera pajang* Kosterm peels. Food Chemistry, 128(4), 1121–1127. 10.1016/j.foodchem.2011.03.105

[fsn3832-bib-0020] Prieto, P. , Pineda, M. , & Aguilar, M. (1999). Spectrophotometric quantitation of antioxidant capacity through the formation of a phosphomolybdenum complex: Specific application to the determination of vitamin E. Analytical Biochemistry, 269, 337–341. 10.1006/abio.1999.4019 10222007

[fsn3832-bib-0021] Rahman, M. , Hossain, S. , Rahaman, A. , Fatima, N. , Nahar, T. , Uddin, B. , & Basunia, M. A. (2013). Antioxidant activity of *Centella asiatica* (linn.) urban: Impact of extraction solvent polarity. Journal of Pharmacognosy and Phytochemistry, 1(6), 27–32.

[fsn3832-bib-0022] Richter, B. E. , Jones, B. A. , Ezzell, J. L. , Porter, N. L. , Avdalovic, N. , & Pohl, C. (1996). Accelerated solvent extraction: A technique for sample preparation. Analytical Chemistry, 68, 1033–1039. 10.1021/ac9508199

[fsn3832-bib-0023] Sahin, S. , & Samli, R. (2013). Optimization of olive leaf extract obtained by ultrasound‐assisted extraction with response surface methodology. Ultrasonics Sonochemistry, 20, 595–602. 10.1016/j.ultsonch.2012.07.029 22964032

[fsn3832-bib-0024] Sies, H. , & Stahl, W. (1995). Vitamins E and C, beta‐carotene, and other carotenoids as antioxidants. American Journal of Clinical Nutrition, 62, 1315–1321. 10.1093/ajcn/62.6.1315S 7495226

[fsn3832-bib-0026] Silva, E. M. , Pompeu, D. R. , Larondelle, Y. , & Rogez, H. (2007). Optimisation of the adsorption of polyphenols from *Inga edulis* leaves onmacroporous resins using an experimental design methodology. Separation and Purification Technology, 53, 274–280. 10.1016/j.seppur.2006.07.012

[fsn3832-bib-0027] Singleton, V. L. , Orthofer, R. , & Lamuela‐Raventos, R. (1999). Analysis of total phenols and other oxidation substrates and antioxidants by means of FC reagent. Methods in Enzymo, 29, 152–178. 10.1016/S0076-6879(99)99017-1

[fsn3832-bib-0028] Song, J. , Li, D. , Liu, C. , & Zhang, Y. (2011). Optimized microwave‐assisted extraction of total phenolics (TP) from Ipomoea batatas leaves and its antioxidant activity. Innovative Food Science and Emerging Technologies, 12, 282–287. 10.1016/j.ifset.2011.03.001

[fsn3832-bib-0029] Srivastava, R. , Shukla, Y. N. , & Kumar, S. (1997). Chemistry and pharmacology of *Centella asiatica*: A review. Journal of Medicine Aromatic Plant Sciences, 19, 1049–1057.

[fsn3832-bib-0300] Stahl, W. , & Sies, H. (2003). Antioxidant activity of carotenoids. Molecular Aspects of Medicine, 24, 345–351. 14585305 10.1016/s0098-2997(03)00030-x

[fsn3832-bib-0030] Şükran, D. E. R. E. , Gunes, T. , & Sivaci, R. (1998). Spectrophotometric determination of chlorophyll‐A, B and total carotenoid contents of some algae species using different solvents. Turkish Journal of Botany, 22(1), 13–18.

[fsn3832-bib-0031] Tabaraki, R. , & Nateghi, A. (2011). Optimization of ultrasonic‐assisted extraction of natural antioxidants from rice bran using response surface methodology. Ultrasonics Sonochemistry, 18, 1279–1286. 10.1016/j.ultsonch.2011.05.004 21612968

[fsn3832-bib-0032] Vita, J. A. (2005). Polyphenols and cardiovascular disease: Effects on endothelial and platelet function. American Journal of Clinical Nutrition, 81, 292–297. 10.1093/ajcn/81.1.292S 15640493

[fsn3832-bib-0033] Wang, L. , & Liu, Y. (2009). Optimization of solvent extraction conditions for total carotenoids in rapeseed using response surface methodology. Natural Science, 1(01), 23 10.4236/ns.2009.11005

[fsn3832-bib-0034] Zainol, M. K. , Abd‐Hamid, A. , Yusof, S. , & Muse, R. (2003). Antioxidative activity and total phenolic compounds of leaf, root and petiole of four accessions of *Centella asiatica* (L.) Urban. Food Chemistry, 81, 575–581.

